# Hypomethylating agents (HMA) for the treatment of acute myeloid leukemia and myelodysplastic syndromes: mechanisms of resistance and novel HMA-based therapies

**DOI:** 10.1038/s41375-021-01218-0

**Published:** 2021-05-06

**Authors:** Julia Stomper, John Charles Rotondo, Gabriele Greve, Michael Lübbert

**Affiliations:** 1 Department of Medicine I, Medical Center - University of Freiburg, Faculty of Medicine, University of Freiburg, Freiburg, Germany; 2 Department of Medical Sciences, University of Ferrara, Ferrara, Italy; 3 German Cancer Research Consortium (DKTK), Freiburg, Germany

**Keywords:** Cancer therapeutic resistance, Myelodysplastic syndrome, Acute myeloid leukaemia

## Abstract

Aberrant DNA methylation plays a pivotal role in tumor development and progression. DNA hypomethylating agents (HMA) constitute a class of drugs which are able to reverse DNA methylation, thereby triggering the re-programming of tumor cells. The first-generation HMA azacitidine and decitabine have now been in standard clinical use for some time, offering a valuable alternative to previous treatments in acute myeloid leukemia and myelodysplastic syndromes, so far particularly in older, medically non-fit patients. However, the longer we use these drugs, the more we are confronted with the (almost inevitable) development of resistance. This review provides insights into the mode of action of HMA, mechanisms of resistance to this treatment, and strategies to overcome HMA resistance including next-generation HMA and HMA-based combination therapies.

## Introduction

Epigenetic modifications, such as DNA methylation represent an important therapeutic target in hematopoietic malignancies [1, 2]. DNA methylation occurs through the covalent addition of a methyl group to the 5′ carbon of the cytosine ring catalyzed by DNA methyltransferases (DNMT), resulting in 5-methylcytosine. There are three members of DNMT that have catalytic activity: DNMT1, DNMT3A, and DNMT3B. DNMT1 is the proposed maintenance methyltransferase that is responsible for copying DNA methylation patterns to the pre-existing hemimethylated post-replication DNA [3]. DNMT3A and DNMT3B are related proteins which establish DNA methylation patterns on unmethylated DNA functioning as de novo methyltransferases [4]. The potential sites within the genome that can be methylated or demethylated are cytosine-guanine dinucleotides referred to as CpG dinucleotides. CpG islands are areas with a high concentration of CpG.

In cancer, aberrant DNA methylation at CpG islands within promoter regions leads to the silencing of critical tumor suppressor genes involved in cancer-related pathways, such as invasion, DNA repair, and cell cycle regulation [1, 2].

## DNA hypomethylating agents

The possibility to induce re-expression of silenced tumor suppressor genes and, in turn, stimulate tumor cells’ re-programming by reversing DNA methylation modifications, led to the pursuit of drugs with hypomethylating potential.

**Fig. 1:**
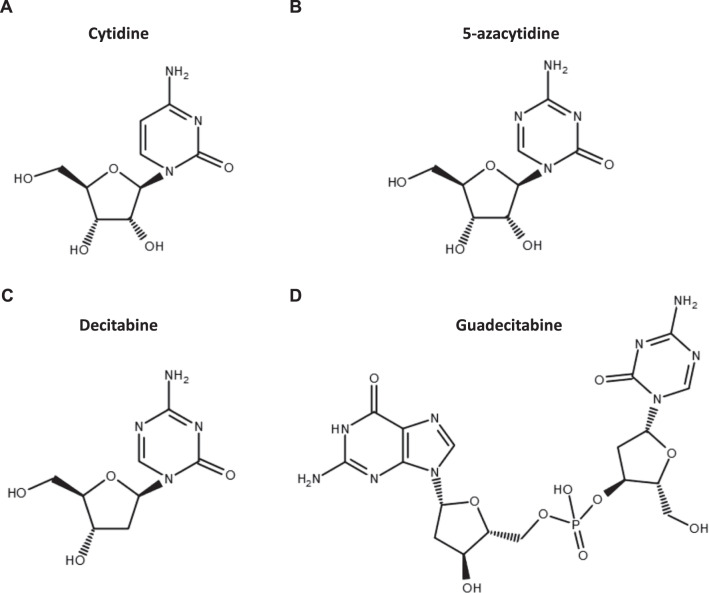
Azanucleoside DNA-hypomethylating agents. Chemical structures of cytidine (**A**), the cytidine analogs 5-azacytidine (**B**) and decitabine (**C**), and guadecitabine (SGI-110), a dinucleotide of decitabine and deoxyguanosine (**D**).

The first generation of DNA hypomethylating agents (HMA) was developed as conventional cytostatic therapy in the 1960s [5]. Administered at high doses, they were found to be too toxic for patients, without having a substantial antitumor effect. More recently, the azanucleosides azacitidine (5-azacytidine) and decitabine (5-aza-2′-deoxycytidine; Fig. 1) were reintroduced at lower and repeated doses: azacitidine administered subcutaneously at a dose of 75 mg/m^2^ for 7 days every 28 days, and decitabine given intravenously at a dose of 15 mg/m^2^ every 8 h for 3 days, repeated every 6 weeks, were shown to have beneficial effects in patients with myelodysplastic syndromes (MDS), which led to their approval by the US Food and Drug Administration (FDA) in 2004 and 2006, respectively, for the treatment of MDS [6, 7]. In addition, a 5-day dosing regimen of decitabine given at 20 mg/m^2^ for 5 days every 28 days, allowing for easier administration in the outpatient setting, was FDA approved in 2010 and has become the clinical standard [8]. Currently, azacitidine and decitabine are broadly used not only for the treatment of MDS but also of older, medically non-fit acute myeloid leukemia (AML) patients [9, 10].

## HMA uptake, metabolism, and mechanism of action

**Fig. 2:**
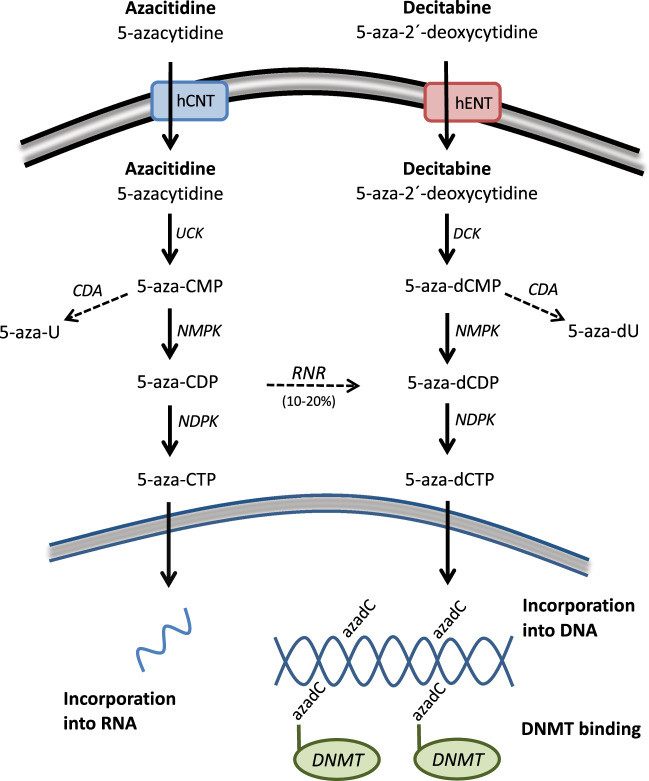
Schematic representation of azacitidine and decitabine uptake and metabolism. 5-aza-U 5-aza-uridine, 5-aza-dU 5-aza-2´-deoxyuridine, CDP cytidine diphosphate, CMP cytidine monophosphate, hCNT human concentrative nucleoside transporter, hENT human equilibrative nucleoside transporter, NDPK nucleoside diphosphate kinase, NMPK nucleoside monophosphate kinase, RNR ribonucleotide reductase.

Azacitidine and decitabine are analogs of the nucleoside cytidine (Fig. 1). The molecular mechanism of action of HMA has been described in detail in a recent review [11]. In brief, it comprises the cellular uptake, intracellular activation, incorporation into nucleic acids, and inhibition of DNMT thereby inducing DNA hypomethylation (Fig. 2). The cellular uptake is mediated by different nucleoside transporters [12, 13]. Three successive phosphorylation events eventually result in the active metabolites 5-azacitidine-triphosphate for azacitidine and 5-aza-2′-deoxycytidine-triphosphate (5-aza-dCTP) for decitabine. The enzymes catalyzing the first limiting phosphorylation step are uridine-cytidine kinase (UCK) for azacitidine and deoxycytidine kinase (DCK) for decitabine (Fig. 2). HMA are considered S-phase-specific drugs because they become incorporated into DNA during replication. While decitabine is exclusively incorporated into DNA, only 10–20% of azacitidine follows the same process, since the majority of azacitidine is incorporated into RNA. Due to the activity of cytidine deaminase (CDA), which can rapidly inactivate cytidine analogs, the half-life of subcutaneous azacitidine and intravenous decitabine in AML/MDS patients is only ~35–40 min [14–16]. In contrast, the half-life of decitabine in buffer at 37 °C and neutral pH is about 10 h [17].

At relatively low doses, incorporated 5-aza-dCTP is recognized and irreversibly bound by DNMT1 [18], inducing the degradation of DNMT1 [19, 20]. The resulting DNA demethylation can lead to the reactivation of aberrantly silenced genes involved in multiple different pathways, such as apoptosis, DNA repair, differentiation, and angiogenesis [11, 21]. Recently, effects on the immune response through activation of endogenous retroviruses have come into focus [22, 23].

## Gene mutations and cytogenetic abnormalities as HMA treatment predictors

In the pursuit of biomarkers to predict responsiveness to HMA in hematologic diseases, somatic gene mutations have been explored in numerous studies. However, as reviewed previously [24], the results obtained thus far have been discordant regarding the predictive and prognostic value of mutations, which is somewhat surprising given the strong biological rationale of considering the mutation status of genes like *DNMT3A* or *TET2*, centrally involved in the regulation of DNA methylation. Possible reasons for the inconsistent results of predictor studies may be, for instance, differences in age groups, gender effects, ethnicity, etc. between different studies [25]. Since only a fraction of patients was commonly affected by mutations in the specific genes under investigation in previous studies, larger cohorts of patients need to be studied to identify molecular predictors of response. In addition, the employment of machine learning approaches might facilitate the identification of genomic biomarkers of response and resistance to HMA, as demonstrated in a recent study including 433 MDS patients [25].

The encouraging (and at first counter-intuitive) response rate of patients with *TP53* mutations [26], often associated with a complex-monosomal karyotype (see below), has important clinical implications. Remissions, even when maintained with continued treatment, are usually of shorter duration than those achieved in patients with wild-type *TP53* (and lack of a complex-monosomal karyotype). The only curative approach, particularly in these patients, often still is provided by allogeneic hematopoietic stem cell transplantation (HSCT) alone. Hence, even in the presence of a hematologic remission, patients bearing *TP53* mutations and eligible for allografting should proceed to the curative approach as soon as possible, i.e., before continued treatment leads to the development of secondary resistance. The modulating role of bi-allelic vs. single *TP53* lesions as predictors of response to HMA treatment and outcome after allografting is the subject of ongoing studies. Also under study are mechanistic aspects of the (albeit transient) response to HMA in these adverse-genetics AML, whether by an interaction of mutated p53 protein with HMA, or by their gene-reactivating effects being particularly attracted by monosomic chromosomes (e.g., chromosome 7) presenting broad epigenetic silencing [27].

Regarding the predictive value of cytogenetic aberrations in MDS and AML patients who receive HMA therapy, a recent study including about 700 patients with higher-risk MDS or low blast count AML found that baseline cytogenetic abnormalities could not predict response to azacitidine treatment [28]. Only in the subgroup of patients with less than 20% bone marrow blasts, 3q abnormalities and complex karyotype were associated with a significantly lower overall response rate. No correlation between hematologic and cytogenetic response was observed in this study. Similarly, another study assessing genetic mutations and cytogenetics in 128 MDS or AML patients treated with azacitidine did not identify a clear biomarker for response or survival [29]. These results are in contrast with previous studies indicating that chromosomal aberrations, in particular abnormalities of chromosome 7, alone or imbedded in complex-monosomal karyotypes, could be predictors of responsiveness to HMA [26, 30, 31]. Further studies are needed to determine the predictive relevance of cytogenetic information for HMA treatment.

## Mechanisms of resistance to HMA

**Fig. 3:**
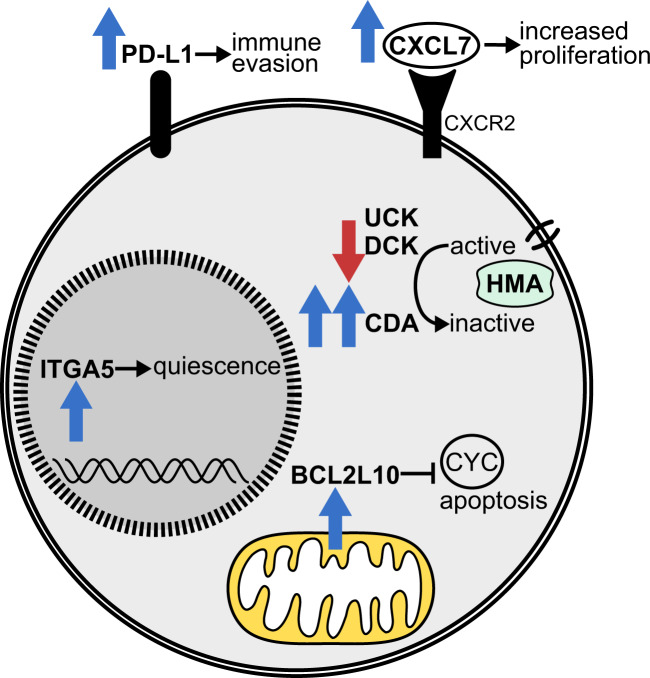
Cell intrinsic factors associated with resistance to HMA therapy in myeloid malignancies. CYC cytochrome c (release), ITGA5 integrin subunit alpha 5.

Despite initial responses to azacitidine and decitabine treatment in a subset of patients with hematologic malignancies, the development of resistance to HMA therapy is an almost inevitable problem, as shown by Prebet et al. for azacitidine already in 2011 [32]. There are two categories of resistance: primary resistance, in which patients do not show any improvement after at least 4–6 cycles of treatment, and secondary resistance, in which initially responding patients relapse after long-term treatment. The exact molecular mechanisms underlying primary or secondary HMA resistance are unknown and different factors have been proposed to be involved, including both mechanisms intrinsic to hematopoietic stem and progenitor cells, and tumor cell extrinsic factors related to immune cells and other cells in the bone marrow milieu (Fig. 3, Table 1).

**Table 1:** Mechanisms and biomarkers associated with resistance to HMA treatment in patients with myeloid malignancies.

	Reference
HMA metabolism	
High CDA/DCK ratio	[36]
Low UCK and DCK expression	[37, 39]
Non-depleted DNMT1	[39]
High SAMHD1 expression	[40]
Cell cycle activity	
High number of quiescent hematopoietic progenitor cells	[41]
Increased integrin α5 signaling	[41]
High CXCL4 and CXCL7 expression	[42]
Genetic and epigenetic mechanisms	
Expansion of resistant subclones	[26, 46]
Differentially methylated regions	[42]
Increase in RNA 5-methylcytosine and NSUN1-/BRD4-associated active chromatin	[44]
Immune response	
Failure to upregulate inflammation-related and immune response gene sets	[41, 48]
High expression of PD-1, PD-L1, PD-L2, and CTLA-4	[65, 66]
Others	
Persistence of leukemia stem and progenitor cells	[47]
High percentage of BCL2L10 expressing bone marrow cells	[49, 50]

### Tumor cell intrinsic factors

At first, various studies focused on alterations related to HMA metabolism, which might impair HMA efficacy and contribute to both primary and secondary resistance. In vitro, resistance to decitabine was shown to be most pronounced in cancer cell lines with low mRNA expression of genes involved in decitabine uptake and activation, and high expression of CDA, the enzyme responsible for the inactivation of cytidine analogs [33]. The same study found that homozygous loss of DCK could cause resistance to decitabine in HL-60 cells. Likewise, mutations in UCK2, the equivalent to DCK in azacitidine metabolism, were shown to cause resistance to azacitidine in vitro by perturbing its activation [34]. Moreover, loss of DCK, UCK2 and the nucleoside transporter ENT1/SLC29A1 has recently been shown to play a role in resistance to azacitidine and guadecitabine in AML cell line and mouse experiments [35].

In contrast to those preclinical findings, HMA resistance in MDS patients could not be clearly linked to impaired drug metabolism thus far. As for decitabine, a subset of MDS patients with primary resistance was shown to have a higher ratio of CDA to DCK, leading to increased inactivation and decreased activation of decitabine, than patients responding to decitabine treatment [36]. The same study investigated mechanisms of secondary resistance to decitabine by comparing diagnosis and relapse samples with respect to mRNA expression levels of genes involved in decitabine metabolism, and the acquisition of DCK mutations. Neither a significant difference in gene expression nor DCK mutations could be detected at relapse. As for azacitidine, a trend towards a lower mRNA expression level of UCK1 was observed in MDS patients without a response compared to patients with a response to azacitidine treatment [37]. Using a novel mass spectrometry method to quantify the active metabolites of azacitidine in MDS and chronic myelomonocytic leukemia (CMML) patients, a recent study, however, concluded that primary resistance to azacitidine was not the result of impaired azacitidine metabolism [38]. Regarding adaptive resistance, another recent study, which analyzed DNMT1 protein levels and expression levels of key pyrimidine metabolism enzymes in serial bone marrow samples from MDS patients receiving HMA treatment, indicated that relapse might be the result of expression changes of pyrimidine metabolism enzymes preventing the depletion of DNMT1 [39]. Finally, pretreatment protein levels of the triphosphohydrolase SAMHD1 in leukemic blasts were recently shown to correlate with response to decitabine but not to azacitidine in AML patients [40]. The selective inactivation of HMA demonstrated in this study was due to the fact that only the active triphosphate metabolite of decitabine but not of azacitidine functions as activator and substrate of SAMHD1.

Due to these conflicting results regarding changes in azanucleoside metabolism as potential causes of resistance, other molecular mechanisms are thought to be involved in HMA resistance. First of all, the cell cycle activity of hematopoietic cells before treatment appears to be critical for azanucleosides to be effective. MDS and CMML patients with primary resistance to azacitidine were found to have more quiescent hematopoietic progenitor cells than patients with response to azacitidine [41]. Cell cycle quiescence was shown to be mediated by integrin α5 signaling, which could potentially be therapeutically exploited by combining azacitidine with an integrin α5 inhibitor to overcome resistance. In CMML patients treated with decitabine, another study found genes associated with the cell cycle to be upregulated at diagnosis in responders compared to non-responders [42]. Non-responders showed overexpression of CXCL4 and CXCL7 in the bone marrow, two chemokines, the former of which regulates the cell cycle activity of hematopoietic stem cells. Their overexpression might contribute to primary decitabine resistance since treatment with CXCL4 and CXCL7 was able to abrogate the effect of decitabine treatment in primary CMML cells [42].

Epigenetic differences between responders and non-responders are another feature related to the mode of action of HMA that has been explored in the context of resistance. DNA methylation differences at baseline, primarily affecting nonpromoter regions, were shown to be predictive of response to decitabine treatment in CMML patients [42]. Assessment of global DNA methylation levels by long interspersed nuclear element-1 (LINE-1) analysis at the time of diagnosis and at relapse has shown that relapse in MDS patients treated with decitabine occurred despite hypomethylation [36]. Conversely, the hematological response of 15 MDS/CMML patients to azacitidine was associated with stable global DNA methylation levels, but significant demethylation of specific CpG of the *EZH2* and *NOTCH1* genes [43]. As for azacitidine, the majority of which is incorporated into RNA, another recent study has demonstrated that certain chromatin structures mediated by RNA cytosine methylation and RNA methyltransferases, including NSUN1, are significantly increased in leukemia cell lines and AML/MDS patients resistant to azacitidine treatment [44].

At the genetic level, several studies showed that malignant clones are not eliminated by hypomethylating treatment, even in patients with complete morphological responses, and that the variant allele frequency remained overall stable [41, 43, 45, 46]. The expansion of resistant subclones, and the population of leukemia stem and progenitor cells not eradicated by epigenetic treatment are therefore potential causes of secondary resistance in patients initially achieving a remission [26, 46, 47].

In vitro studies demonstrating the stimulation of an antiproliferative immune response by induction of endogenous retroviruses in malignant cells provided first evidence that inflammation and immune response pathways are involved in the mode of action of HMA [22, 23]. A recent study including 40 patients with different hematologic disorders treated with azacitidine suggested that these pathways play a role also in vivo, as the induction of evolutionary young transposable elements and the activation of the innate immune system were observed in responders [48]. Similarly, another study found that inflammation and immune response pathways were upregulated in hematopoietic progenitor cells of MDS and CMML patients sensitive to azacitidine treatment whereas an alteration of those pathways was not observed in non-responders [41].

Lastly, BCL2L10, an anti-apoptotic member of the B-cell lymphoma-2 (BCL-2) family, has been proposed as a biomarker for response to azacitidine and overall survival (OS) in AML/MDS patients. Results of a retrospective study showing a correlation between an increased percentage of BCL2L10 expressing bone marrow cells and azacitidine resistance have been confirmed prospectively [49, 50].

### Effects of HMA on immune cells (tumor cell extrinsic factors)

In addition to their direct effects on the malignant clone in AML/MDS, HMA also demonstrate effects on cells of the immune system and the bone marrow niche (Table 2). Indeed, the enormous potential of (re)activation of an immune-mediated antitumor response has been recognized through studies of in vitro and in vivo effects of HMA over the last decade (comprehensively reviewed by Jones and colleagues [51]).

**Table 2:** Effects of HMA therapy on different immune cells in the murine system (M) and in humans (H).

Cell type	HMA effect	Reference
T cells	Induction of CD8^+^ T-cell responses to tumor antigens (H)	Goodyear et al. [55]
	Increase in IFN-gamma^+^ T cells (H)	Li et al. [122]
	Enhanced CD8^+^ T-cell response by upregulation of MHC-1 (M, H)	Luo et al. [123]
	Improvement of T-cell frequency and repertoire in MDS (H)	Fozza et al. [124]
	Reversion of exhaustion-associated de novo methylation programs → rejuvenation of exhausted CD8^+^ T cells after sequential DAC and anti-PD-L1 treatment (M)	Ghoneim et al. [53]
Regulatory T cells (Tregs)	Expansion of Tregs after allo HSCT (M, H) and in autoimmune disease (rodent model)	Sánchez-Abarca et al. [54]
		Goodyear et al. [55]
		Cooper et al. [125]
	Reduction in number and function of Tregs in MDS (H, in vitro HMA treatment)	Fagone et al. [126]
		Costantini et al. [127]
Natural killer cells	Induction of KIR expression (H, cell lines)	Santourlidis et al. [128]
		Sohlberg et al. [57]
	Decrease or increase in NK cell functionality and number (H, M, cell lines)	Gao et al. [129]
		Schmiedel et al. [58]
		Kübler et al. [56]
	Increased susceptibility of AML blasts to anti-CD33 antibody and NK-mediated ADCC (H)	Vasu et al. [59]
Dendritic cells	Increased CD40 and CD86 expression (H)	Frikeche et al. [130]
	Decreased IL-10 and IL-27 secretion (H)	Kwon et al. [60]
	Activation and increase in IFN-gamma levels (M)	
Myeloid-derived suppressor cells	Decrease in cell number (M)	Triozzi et al. [61]
		Kim et al. [62]
		Luker et al. [63]
		Zhou et al. [131]
Mesenchymal stromal cells (MSCs)	Increased support of healthy over clonal (MDS) hematopoietic stem and progenitor cell expansion (H; coculture conditions)	Wenk et al. [64]
	Decrease in IL-6 production in MSCs from MDS patients to levels found in normal controls (H, in vitro AZA treatment)	Boada et al. [132]
	Increased immunomodulation and migration (M, human cells)	Lee et al. [133]

*ADCC* antibody-dependent cellular cytotoxicity, *AZA* azacitidine, *C* cell lines, *H* in humans, *KIR* killer cell immunoglobulin-like receptors, *M* in the murine system.

In a very recent study, systematic, serial in vivo profiling by flow cytometry was performed on different T-cell subpopulations of AML patients before and after decitabine treatment, showing an increase of CD38 expression on CD8^+^ and CD4^+^ T cells upon decitabine treatment [52]. Interestingly, CD38 expression on CD8^+^ T cells was negatively correlated with interferon-gamma production by CD8^+^ T cells in this study, indicating decreased T-cell function in this model. As regards CD8^+^ T-cell rejuvenation by exposure to an HMA, Ghoneim et al. have recently demonstrated that reversion of exhaustion-associated de novo methylation programs in CD8^+^ T cells was observed after sequential decitabine and anti-programmed death ligand 1 (PD-L1) treatment [53].

The induction of regulatory T cells by pharmacological demethylation of the *FOXP3* promoter using HMA, first described by Sánchez-Abarca et al. [54] and Goodyear et al. [55], is by now well-established. Furthermore, various groups have investigated the effects of HMA on the number and function of natural killer (NK) cells, with the majority of studies demonstrating an activating effect [56, 57]. One study reported differential effects of the two HMA, with decitabine augmenting NK cell responsiveness towards stimulation, and, in contrast, azacitidine impairing NK cell reactivity [58]. Moreover, decitabine has been shown to increase the susceptibility of AML blasts to anti-CD33 antibody and NK-mediated antibody-dependent cellular cytotoxicity [59].

Regarding dendritic cells, a clinically very relevant recent study, utilizing a murine graft-versus-leukemia model, demonstrated that decitabine is able to prime allogeneic immune reactions of donor lymphocyte infusions (DLI) by activating dendritic cells, also via HMA-induced increase in interferon-gamma levels [60].

In contrast, myeloid-derived suppressor cells have been shown to be reduced upon HMA treatment in tumor-bearing mice [61–63]. The function of cells of the bone marrow microenvironment, such as mesenchymal stromal cells (MSC) can also be affected by HMA treatment, as e.g., shown for MSC from MDS patients [64].

In aggregate, these various experimental approaches demonstrate that HMA are able to trigger or boost non-self recognition and cytotoxic T-cell activity against malignant cells, and reactivate interferon-response genes, also by reactivating endogenous retroviruses resulting in “viral mimicry” via enhanced interferon-gamma response (see also [22, 23]).

With the advent of therapeutic antibodies reactivating immune checkpoints, HMA treatment has also been shown to upregulate the expression of the inhibitory immune checkpoint receptors programmed death 1 (PD-1) and cytotoxic T lymphocyte-associated antigen 4 (CTLA-4) in T cells, and their ligands PD-L1 and PD-L2 in tumor cells, respectively. MDS, CMML, and AML patients resistant to epigenetic therapy have been reported to show a trend towards a higher relative increase in PD-1, PD-L1, PD-L2, and CTLA-4 gene expression than patients who achieved a response [65]. Increased PD-1 expression upon HMA treatment has been shown to be related to PD-1 promoter demethylation in both leukemia cell lines and patient T-cell samples [65, 66], and demethylation of the PD-1 promoter to be associated with a lower response rate to azacitidine in AML/MDS [66]. Thus, upregulation of the expression of inhibitory immune checkpoint receptors and their ligands might result in secondary resistance to HMA treatment.

## Strategies to overcome HMA resistance

HMA are broadly used in the treatment of AML and MDS. However, despite their generally accepted (albeit temporary) efficacy, the major limitation of this treatment lies in primary or secondary resistance. The rate of relapse after azacitidine treatment observed from clinical trials in MDS patients has been reported to be 36% [32]. The problem of secondary resistance raises the question of switching one HMA to another to regain drug sensitivity. While early anecdotal clinical evidence pointed to the strategy of switching from decitabine to azacitidine or vice versa in case of secondary resistance [67], subsequent retrospective studies demonstrated little benefit of such an approach, and it has not been adopted in clinical practice [68, 69]. However, no randomized trial has compared this approach. Encouraging recent results from Gu et al. indicate that alternating treatment may overcome adaptive responses of the pyrimidine metabolism network, and in a mouse model has resulted in significant survival extension when both HMA were combined with the CDA inhibitor tetrahydrouridine [39]. Recent studies and clinical trials have explored novel HMA, modified treatment schedules, and combination treatments with an HMA backbone as potential strategies to overcome HMA resistance (Table 3).

**Table 3:** Examples of novel HMA-based treatment regimens for AML and MDS.

Treatment regimen	Route of administration of novel HMA or combination agent	Mechanism of action of novel HMA or combination agent	Current status of development	Reference/NCT number
*Novel HMA*				
Guadecitabine (SGI-110)	s.c.	Resistance to the DAC-degrading enzyme CDA resulting in extended exposure to DAC	Phase 3 trials	[73, 74], NCT02907359, NCT02920008
ASTX727 (cedazuridine/DAC)	oral	CDA inhibition	FDA approved	[75–77]
CC-486	oral	Oral formulation of AZA, allowing for extended lower drug exposure	FDA approved	[79, 81], NCT04173533
*Combination regimens with an HMA backbone*				
HMA + venetoclax	oral	BCL-2 inhibition	FDA approved	[90]
DAC + ATRA	oral	Induction of differentiation	Phase 2 trial	[94]
HMA + HDAC inhibitor	oral	Gene reactivation	Phase 2 trials	[83, 94, 99, 100]
HMA + PD-1, PD-L1, or CTLA-4 inhibitor	i.v.	Immune checkpoint blockade	Phase 1/2 trials	[101]
AZA + magrolimab	i.v.	Anti-CD47 antibody inhibiting a macrophage immune checkpoint	Phase 1/3 trials	[102], NCT03248479, NCT04313881
HMA + lenalidomide	oral	Immunomodulation	Phase 2/3 trials	[103, 104]
DAC + bortezomib	s.c.	Proteasome inhibition	Phase 2 trial	[105]
DAC + ibrutinib	oral	BTK inhibition	Phase 2 trial	[106]
HMA + FLT3 inhibitor	oral	Tyrosine kinase inhibition	Phase 2/3 trials	NCT02752035, NCT04097470,
HMA + ivosidenib or enasidenib	oral	IDH1/2 inhibition	Phase 2/3 trials	NCT03173248, NCT02677922, NCT03683433, NCT03383575
AZA + APR-246	i.v.	Mutant p53 activation	Phase 2/3 trials	[110], NCT03745716
AZA + rigosertib	oral	Multikinase inhibition	Phase 1/2 trial	[113]
AZA + pevonedistat	i.v.	NEDD8-activating enzyme inhibition	Phase 2 trial	[114]

*AZA* azacitidine, *BTK* Bruton’s tyrosine kinase, *DAC* decitabine, *i.v.* intravenous, *s.c.* subcutaneous.

### Novel HMA including oral formulations

Guadecitabine (SGI-110), a dinucleotide of decitabine and deoxyguanosine (Fig. 1), is a next‐generation subcutaneous HMA, which is resistant to CDA, the main enzyme responsible for decitabine degradation. The half-life of the active metabolite decitabine is longer after subcutaneous administration of guadecitabine than after intravenous administration of decitabine [70]. The extended exposure to decitabine might result in increased drug effectiveness as the incorporation of decitabine into DNA is cell cycle dependent. Guadecitabine activity has been demonstrated in phase 1/2 studies in both treatment-naive and relapsed or refractory AML and MDS patients [70–72]. While the results of a phase 3 trial of guadecitabine vs. treatment choice in MDS and CMML patients previously treated with azacitidine and/or decitabine are not yet available (NCT02907359), the large randomized phase 3 trial ASTRAL-1 showed that guadecitabine was not superior to treatment choice with regard to complete remission rate and OS in treatment-naive AML patients unfit for intensive chemotherapy [73]. An additional analysis of this trial reported a survival benefit from guadecitabine as compared to treatment choice in the subgroup of patients who received at least four or six cycles [74]. In patients who received at least four cycles, median OS was 15.6 months for the guadecitabine and 13 months for the treatment choice group (hazard ratio, 0.78; 95% confidence interval, 0.64–0.96); in patients who received at least six cycles, median OS was 19.5 and 15 months (hazard ratio, 0.69; 95% confidence interval, 0.54–0.88), respectively.

The novel oral HMA ASTX727 consists of a fixed-dose combination of decitabine at 35 mg and the CDA inhibitor cedazuridine at 100 mg. Cedazuridine is able to increase the oral bioavailability of decitabine by limiting its rapid CDA-mediated degradation in the gut and liver. In fact, by adding cedazuridine to oral decitabine, systemic decitabine exposure has been shown in phase 1–3 studies to be equivalent to that after intravenous administration of decitabine [75–77]. Randomized phase 2 and 3 trials additionally demonstrated similar DNA demethylation measured by LINE-1 assays and clinical efficacy between cedazuridine/decitabine and intravenous decitabine in MDS and CMML patients [76, 77]. Given the equivalent systemic decitabine exposure, those results were not unexpected. In July 2020, the FDA approved the oral combination of decitabine and cedazuridine for the treatment of MDS and CMML.

Apart from its successful combination with decitabine, the novel CDA inhibitor cedazuridine has also been studied in combination with CC-486, an oral formulation of azacitidine. In animal models, the combination of oral azacitidine and cedazuridine was shown to have similar bioavailability as parenteral azacitidine [78]. Moreover, oral azacitidine and cedazuridine as well as an oral triple therapy comprising this combination plus venetoclax resulted in decreased leukemic expansion in an AML patient-derived xenograft model [78].

Single treatment with the oral azacitidine formulation CC-486 has been investigated in different clinical settings. Taking advantage of the ease of administration and the potential benefit of extended lower drug exposure with oral azacitidine, CC-486 has been proposed as postintensive chemotherapy or posttransplant maintenance therapy in AML and MDS [79, 80]. In the phase 3, randomized, placebo-controlled QUAZAR AML-001 trial, CC-486 at a dose of 300 mg once daily on days 1–14 of 28-day cycles significantly improved OS and relapse-free survival in older AML patients who were in first remission after intensive chemotherapy and not candidates for allogeneic HSCT [79]. In the posttransplant setting, encouraging results were obtained with a 14-day dosing schedule of CC-486 maintenance therapy in a phase 1/2 dose-finding study in AML/MDS patients in remission after HSCT [80], and this concept is being further evaluated in an ongoing phase 3 trial (NCT04173533). Lastly, CC-486 might be a treatment option for lower-risk MDS patients with transfusion-dependent anemia and thrombocytopenia as shown in a recent randomized, placebo-controlled phase 3 trial [81].

### Modified HMA dosing to enhance antileukemic activity

Given the mechanism of action of HMA, with the induction of hypomethylation after S-phase dependent incorporation into DNA, alternative dosing schedules have been proposed as a way to enhance the antileukemic activity of HMA. An intensified dosing regimen of parenteral azacitidine at 75 mg/m^2^ given for 5 days every 14 days for four cycles, which increased the number of days of azacitidine treatment during the first 8 weeks of treatment by 18%, showed promising results in terms of early response rate, OS, and hematologic toxicity in a study of 26 higher-risk MDS patients [82]. A phase 2 study found that prolonged administration of subcutaneous azacitidine at a lower daily dose (50 mg/m^2^ given for 10 days every 28 days) increased the rate of hematologic normalization in MDS and AML patients compared to standard dosing in the reference Cancer and Leukemia Group B 9221 trial [83]. A 14- or 28-day schedule of the oral azacitidine formulation CC-486 has been shown to induce sustained DNA hypomethylation over 28-day treatment cycles [84]. In the phase 3, placebo-controlled QUAZAR lower-risk MDS trial, the extended dosing regimen of CC-486 at 300 mg for 21 days of 28-day cycles significantly reduced transfusion requirements but was associated with an increased incidence of adverse events and early deaths, indicating that the extended schedule might not be broadly applicable [81]. As mentioned above, the shorter 14-day dosing schedule of CC-486 has been proposed as maintenance therapy after intensive chemotherapy or allogeneic HSCT.

Several studies with an intensified schedule of decitabine (20 mg/m^2^ given on days 1–10 of 28-day cycles instead of on days 1–5) have shown an improved response rate in AML/MDS patients [26, 85, 86]. Based on these studies, the clinical activity of the next-generation HMA guadecitabine is under evaluation with 5- and 10-day dosing regimens. No difference in efficacy between those dosing schedules was observed in elderly, medically non-fit treatment-naive AML patients [71]. The 5-day regimen was administered in a global randomized phase 3 trial comparing guadecitabine against treatment choice in 815 treatment-naive AML patients (ASTRAL-1). As described above, this largest trial ever conducted in elderly non-fit AML patients demonstrated that guadecitabine is an active drug, and, while showing an overall similar efficacy and safety profile as standard therapy, appears to develop superiority when administered for at least 4–6 treatment cycles [73, 74].

### Combination treatments with an HMA backbone

Combination therapies with an HMA backbone are increasingly being studied as novel agents become available for the treatment of AML/MDS, and some of them have been shown to have an effect on treatment resistance (Table 3). The combination of HMA with the BCL-2 inhibitor venetoclax is a most promising treatment approach, which has been adopted at many centers and may result in a higher depth of response and treatment prolongation by delaying resistance. Whereas HMA monotherapy fails to eradicate leukemia stem cells [47], BCL-2 inhibition has been shown to selectively eradicate leukemia stem cells by suppressing oxidative phosphorylation [87], explaining the favorable clinical activity of the combination treatment of azacitidine and venetoclax in older AML patients [88]. Moreover, transcriptional induction of the proapoptotic BH3-only protein NOXA by azacitidine has been reported as a novel mechanism of the combinatorial activity of azacitidine and venetoclax [89]. The randomized, placebo-controlled, phase 3 trial VIALE-A evaluated the combination of azacitidine plus venetoclax in newly diagnosed older AML patients ineligible for intensive therapy. In this study, improved median OS (14.7 months vs. 9.6 months; hazard ratio for death, 0.66; 95% confidence interval, 0.52–0.85), more rapid and more durable responses, as well as an increased incidence of transfusion independence were observed with the combination treatment [90]. Responses to the combination therapy were observed across different risk and molecular subgroups including patients with secondary AML or *TP53* mutation.

Despite these promising results, resistance to venetoclax combination therapy constitutes an emerging problem in clinical practice and a few potential mechanisms of resistance have been revealed. A recently published study compared the molecular patterns of patients with response or resistance to venetoclax-based therapy in AML patients [91]. Primary or adaptive resistance to venetoclax plus HMA or low-dose cytarabine were associated primarily with *FLT3*-ITD, other kinase activating mutations, and *TP53* alterations. Another study found that monocytic AML was more resistant to azacitidine plus venetoclax treatment than earlier developmental stages due to a loss of BCL-2 expression [92]. Pharmacologic inhibition of mitochondrial translation leading to the activation of a cellular stress response was recently demonstrated as a potential mechanism to overcome venetoclax resistance in AML [93]. Resistant AML cells were shown to be particularly sensitive to the triple combination of a ribosome-targeting antibiotic like tedizolid, venetoclax, and azacitidine, which might be a treatment regimen worth testing in future clinical trials.

Very recently, decitabine in combination with all-*trans* retinoic acid (ATRA) was shown to result in a statistically significant and clinically meaningful survival extension compared to decitabine alone in elderly non-fit AML patients treated within the phase 2 DECIDER trial [94]. The results of this study suggested that the particular clinical activity of this combination may be due to the delay of resistance development. Clinical efficacy of the decitabine plus ATRA combination regimen in elderly AML patients has also been shown in two single-center studies [95, 96]. Cao et al. additionally performed in vitro studies and found a synergistic antineoplastic effect of decitabine plus ATRA on AML cells with modulation of the miR-34alpha/MYCN axis as a potential underlying mechanism [95]. Exceptional responses to the triple combination of azacitidine, ATRA, and pioglitazone were first reported in a small series of chemorefractory AML patients [97]. Moreover, this combination treatment has been shown to induce myeloid differentiation of primary AML blasts [98].

The addition of the histone deacetylase inhibitors (HDACi) entinostat, valproic acid, or vorinostat to HMA treatment has been investigated in several phase 2 clinical trials, but none of them could show an increased benefit of the combination regimen compared to HMA monotherapy in AML/MDS patients [83, 94, 99, 100]. Possible reasons why the combination of HMA and HDACi—despite the strong biological rationale of a two-pronged approach to relieve epigenetic silencing by inhibition of DNMT and HDAC—so far has not been successful in the clinic, may include additive myelotoxicity; also, simultaneous administration of both drugs may result in G_1_ arrest (induced by HDACi), which in turn may decrease the rate of incorporation of the HMA into DNA. For valproic acid, the serum levels achieved clinically with continued oral administration (comparable to those attained in neurological patients) may be insufficient to result in antileukemic activity.

The therapeutic role of the combination of HMA with immune checkpoint inhibitors (PD-1, PD-L1, and/or CTLA-4 inhibitors) in AML and MDS, which has been discussed in a recent review [101], is currently being evaluated in a number of ongoing phase 1/2 clinical trials. Moreover, several novel anti-CD47 antibodies such as magrolimab, which inhibit a macrophage immune checkpoint, are under study in combination with azacitidine (NCT03248479, NCT04313881) [102].

Lenalidomide, approved by the FDA for the treatment of MDS, has been evaluated in combination with azacitidine in AML and MDS. Randomized studies could not demonstrate an advantage of the combination regimen over azacitidine monotherapy [103, 104]. Similarly, negative results were recently reported for the combination of 10-day decitabine and the proteasome inhibitor bortezomib in AML patients [105], and for 10-day decitabine in combination with the Bruton’s tyrosine kinase inhibitor ibrutinib in AML and MDS patients [106].

Targeted agents, such as FLT3 or IDH inhibitors, and the mutant p53 activator APR-246 constitute another group of drugs which is under investigation in combination with HMA. The combination of HMA and FLT3 inhibitor in *FLT3*-mutated unfit AML patients has been evaluated in several phase 1/2 studies, and a randomized phase 3 trial testing the combination of azacitidine with the FLT3 inhibitor gilteritinib is currently ongoing (NCT02752035). In addition, intensified treatment regimens, such as 10-day decitabine plus midostaurin (NCT04097470), or triple combinations, e.g., decitabine, venetoclax and quizartinib [107], or azacitidine, venetoclax and gilteritinib (NCT04140487) might improve treatment efficacy and are also being explored in clinical trials.

As for IDH inhibitors, different combination therapies with HMA are currently under evaluation in phase 2 and 3 trials. A randomized, placebo-controlled, phase 3 trial tests the combination of azacitidine and ivosidenib in previously untreated *IDH1*-mutated AML (NCT03173248). Phase 2 trials investigate the combination of azacitidine and enasidenib in newly diagnosed and recurrent or refractory IDH2-mutated AML (NCT02677922, NCT03683433) [108], as well as in *IDH2*-mutated MDS (NCT03383575). The phase 3 IDHENTIFY trial, comparing enasidenib with conventional care regimens in patients with relapsed/refractory AML, was recently reported to have failed to meet the primary endpoint OS (NCT02577406). Analogous to the triple combination of HMA, venetoclax and FLT3 inhibitor as targeted agent for *FLT3*-mutated AML, the combination of azacitidine, venetoclax, and ivosidenib is under investigation for *IDH1*-mutated hematologic malignancies in an early-phase clinical trial (NCT03471260) [109].

Combined with azacitidine, the mutant p53 activator APR-246 has shown promising clinical activity in *TP53*-mutated MDS and AML patients in a phase 2 study [110]. The FDA has granted breakthrough therapy designation to this combination regimen for *TP53*-mutated MDS patients, which is further being evaluated in a randomized phase 3 trial (NCT03745716). Moreover, the combination of APR-246 and azacitidine is under investigation as maintenance therapy after allogeneic HSCT in AML and MDS patients with *TP53* mutation (NCT03931291), and as part of a triple therapy with venetoclax in a phase 1 study in patients with *TP53*-mutated hematologic malignancies (NCT04214860).

Rigosertib, an inhibitor of the RAS-RAF-MEK and phosphatidylinositol 3-kinase pathways [111], was previously evaluated as single-agent treatment in high-risk MDS patients after HMA failure [112]. Negative results were recently reported from the phase 3 INSPIRE study on single-agent rigosertib in MDS patients after HMA treatment failure (NCT02562443). Regarding the combination of oral rigosertib and parenteral azacitidine, phase 1 results of a phase 1/2 study in MDS and AML patients showed a safety profile similar to single-agent azacitidine and responses in 7/9 MDS/CMML patients and 2/7 AML patients [113]. Another novel drug that is under active investigation in combination with azacitidine is the NEDD8-activating enzyme inhibitor pevonedistat. In a recent randomized phase 2 study, the combination treatment compared favorably with azacitidine monotherapy in higher-risk MDS patients [114].

Lastly, the combination of HMA with standard chemotherapy is not to be neglected in the context of resistance. The concept of epigenetic priming with decitabine prior to standard induction chemotherapy has proved to be feasible in a phase 1 study of AML patients [115]. Recently, the combination of HMA and standard chemotherapy has been demonstrated in preclinical models to prevent the development of chemoresistant AML relapses through a decrease in AML clones with stemness properties like quiescence and leukemia-initiating capacity [116], providing a rationale for further research on this combination therapy.

Finally, the addition of HMA to DLI, a combination regimen reserved for patients having undergone allogeneic HSCT, can be safe and effective in relapsed AML and MDS patients [117, 118]. Interestingly, the combination of HMA and DLI may enhance the graft-versus-leukemia effect of the donor lymphocytes. The optimal timing as well as DLI dosing are not yet determined, and as shown by Kwon et al. [60], the specific timing of serial dosing may enhance the graft-versus-leukemia effect in a mouse model, while at the same time reducing graft-versus-host disease.

Regarding the feasibility of combinatorial approaches with an HMA backbone, the effects of additive myelotoxicity have to be taken into account, particularly in elderly, medically non-fit AML/MDS patients. Thus, the experience so far demonstrates that even with well-tolerated single-agent administration of an antibody conjugate (e.g., the anti-CD33 antibody vadastuximab talirine, SGN-CD33A) [119], the combination with an HMA may lead to unacceptable myelotoxicity [120]. A similar experience exists for the Polo-like kinase 1 inhibitor volasertib, demonstrating promising results in combination with low-dose cytarabine [121], but necessitating re-evaluation of dose and safety profile when combined with HMA. On the other hand, non-myelotoxic combination partners, such as ascorbic acid or retinoic acid are less likely to result in aggravation of the hematologic toxicity profile of HMA.

## Conclusions and outlook

Dysregulated DNA methylation is a key event in tumor initiation and progression. Targeting DNA methylation using HMA has been a major advance in the treatment of several myeloid neoplasms. The first generation of HMA, i.e., azacitidine and decitabine, has become a cornerstone of MDS and AML treatment. The improved understanding of the mode of action of these agents, together with increasing knowledge about mechanisms of resistance, can inform the selection of patients for HMA therapy, as well as the development of novel HMA and combination treatments with an HMA backbone. Triple combinations including also BCL-2 inhibition or other targeted agents, as well as HMA maintenance therapy are research areas under active investigation. As more and more therapeutic options become available for the treatment of MDS and AML, the sequential use of therapies might be an alternative to combination regimens, and also help to overcome treatment resistance. In order to improve HMA efficacy, studies of new treatment approaches include research on predictive biomarkers.
